# COVID-19 Vaccine Administration, by Race and Ethnicity — North Carolina, December 14, 2020–April 6, 2021

**DOI:** 10.15585/mmwr.mm7028a2

**Published:** 2021-07-16

**Authors:** Charlene A. Wong, Shannon Dowler, Amanda Fuller Moore, Erin Fry Sosne, Hayley Young, Jessica D. Tenenbaum, Cardra E. Burns, Sydney Jones, Marina Smelyanskaya, Kody H. Kinsley

**Affiliations:** ^1^North Carolina Department of Health and Human Services; ^2^Resolve to Save Lives, New York, New York.

COVID-19 has disproportionately affected non-Hispanic Black or African American (Black) and Hispanic persons in the United States (*1*,*2*). In North Carolina during January–September 2020, deaths from COVID-19 were 1.6 times higher among Black persons than among non-Hispanic White persons (*3*), and the rate of COVID-19 cases among Hispanic persons was 2.3 times higher than that among non-Hispanic persons (*4*). During December 14, 2020–April 6, 2021, the North Carolina Department of Health and Human Services (NCDHHS) monitored the proportion of Black and Hispanic persons* aged ≥16 years who received COVID-19 vaccinations, relative to the population proportions of these groups. On January 14, 2021, NCDHHS implemented a multipronged strategy to prioritize COVID-19 vaccinations among Black and Hispanic persons. This included mapping communities with larger population proportions of persons aged ≥65 years among these groups, increasing vaccine allocations to providers serving these communities, setting expectations that the share of vaccines administered to Black and Hispanic persons matched or exceeded population proportions, and facilitating community partnerships. From December 14, 2020–January 3, 2021 to March 29–April 6, 2021, the proportion of vaccines administered to Black persons increased from 9.2% to 18.7%, and the proportion administered to Hispanic persons increased from 3.9% to 9.9%, approaching the population proportion aged ≥16 years of these groups (22.3% and 8.0%, respectively). Vaccinating communities most affected by COVID-19 is a national priority (*5*). Public health officials could use U.S. Census tract-level mapping to guide vaccine allocation, promote shared accountability for equitable distribution of COVID-19 vaccines with vaccine providers through data sharing, and facilitate community partnerships to support vaccine access and promote equity in vaccine uptake.

On January 14, 2021, NCDHHS adopted an equity-promoting vaccination strategy that included both per capita and equity-based vaccine allocation, shared accountability, and community partnerships. Approximately 20% of COVID-19 vaccine doses were set aside for allocation informed by mapping. NCDHHS mapped the proportion of Black and Hispanic populations aged ≥65 years, by U.S. Census Bureau tracts using ArcGIS (version 10.8.1; Esri), and overlayed vaccine provider locations, including Federally Qualified Health Centers (FQHCs).^†^ During February 1–21, 2021, North Carolina received 359,225 (76.3%) of 470,825 mRNA COVID-19 vaccine first dose allocations, distributed to all 100 counties across the state proportional to the total population; 111,600 (23.7%) were directed to census tracts with the highest population proportion of Black, Hispanic, or American Indian or Alaska Native (AI/AN) persons aged ≥65 years. During February 22–March 22, 2021, maps were updated weekly to identify census tracts in which a larger proportion of Black, Hispanic, or AI/AN persons aged ≥65 years remained unvaccinated.^§^ Based on this mapping and vaccine supplies, the decision was made to allocate 20,800 additional doses per week for these census tracts. Doses allocated based on mapping were directed to vaccine providers with geographic proximity to priority census tracts or vaccine providers who served Black or Hispanic populations, such as FQHC.

NCDHHS advised vaccine providers about administering doses to match or exceed their local population proportion of Black and Hispanic persons aged ≥16 years to promote shared accountability with providers for equitable distribution of COVID-19 vaccines (Table). NCDHHS required that vaccine providers report race and ethnicity for each vaccine recipient and provided performance reports twice per month showing the provider-specific vaccine administration to each racial and ethnic group aged ≥16 years relative to population proportions. Statewide and county-specific vaccination data were also published on a dashboard, by race and ethnicity.^¶^ In addition, NCDHHS facilitated two or three meetings per week with providers to exchange best practices and provided tailored coaching on implementing these practices. Community partnerships were facilitated with vaccine providers and trusted messengers in faith- and community-based organizations to support vaccine access. For example, NCDHHS created vaccination and communications toolkits for community-based organizations that included checklists, presentations, and testimonials. NCDHHS also distributed a list of organizations interested in supporting vaccination events to vaccine providers.** This activity was reviewed by CDC and was conducted consistent with applicable federal law and CDC policy.^††^

**TABLE:** Ten strategies recommended by North Carolina Department of Health and Human Services to promote equitable distribution of vaccines, by domain — North Carolina, December 14, 2020–April 6, 2021

Domain	Strategy
**Tailor efforts**	1. Hold appointment slots for underserved populations. For example, reserve 40 out of 100 appointments based on community demographics to ensure these slots are filled with patients from underrepresented communities first. Note this on waiting lists or create different waiting lists to allow for this prioritization. Preferentially reach out to patients from underrepresented communities and schedule these slots before opening appointments to the general population.
	2. Partner with subsidized housing organizations and offer on-site vaccination events with appointments planned and scheduled with housing partner.
	3. Partner with trusted messengers in faith and other community organizations, including those that cater to seniors.*
**Mitigate barriers to accessing web-based scheduling systems**	4. Print and prepopulate event tickets with time and date of vaccine slot; distribute in person to groups who meet the priority criteria; allow them to transfer their ticket to someone else who meets criteria in their place.
	5. Ask partner organization to assist with scheduling appointments, conducting targeted outreach via phone or in person. If working with one partner or more, allow each partner organization to reserve a set number of slots to fill with prioritized populations.
	6. Educate partners to serve as “vaccine ambassadors” to conduct outreach and let eligible groups know how to sign up for a vaccine appointment.
**Mitigate physical and perceived barriers**	7. Host vaccination event at a location that is easy to access through public transportation and familiar to participants.
	8. When registering participants, ask how the person intends to travel to the site and help arrange and/or subsidize transport, if needed.
	9. Extend vaccine event hours to the evenings and weekends to accommodate persons who are unable to take time off from work or those requiring transport from family members.
	10. Do not request photo identification or proof of residency to be vaccinated or to schedule an appointment. The need for an identification card might be a barrier for many populations, including older adults, immigrants, and persons experiencing homelessness.

*Examples include Meals on Wheels, local offices or councils on aging, Association for Home & Hospice Care of North Carolina, LATIN-19, AME Zion Church, North Carolina Rural Coalition Fighting COVID-19, and Rural Forward NC.

During December 14, 2020–April 6, 2021, the number and percentage of vaccine doses allocated based on mapping were calculated. The number and proportion of vaccinated persons were calculated by race and ethnicity with corresponding 95% confidence intervals (CIs). The focus of this analysis was on Black and Hispanic populations.^§§^ For each week during December 14, 2020–April 6, 2021, the proportions of vaccinated persons aged ≥16 years who were Black or Hispanic were compared with the population proportions of Black and Hispanic persons aged ≥16 years statewide, by county and provider type (e.g., FQHC).^¶¶^ Changes over time were assessed by testing differences in proportions (z-statistic).

Among 3,185,750 COVID-19 vaccine doses allocated by NCDHHS during December 14, 2020–April 6, 2021, 226,900 (7.1%) were allocations based on mapping that were directed to 324 vaccine providers in 80 counties, including 83 FQHC (Figure 1). FQHC received 37,900 (16.7%) equity-based allocations.

**FIGURE 1:**
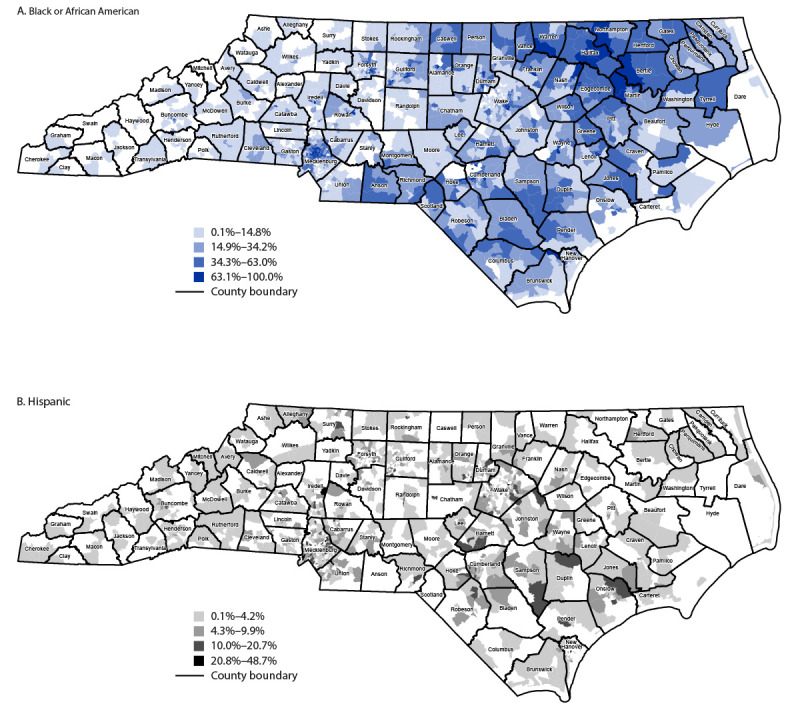
Proportion of Black or African American (A) and Hispanic (B) persons aged ≥65 years, by U.S. Census tract* — North Carolina, 2019† * County boundaries are approximate. † Data from U.S. Census Bureau American Community Survey (2019 5-year estimates).

During December 14, 2020–April 6, 2021, a total of 2,815,774 persons aged ≥16 years received ≥1 vaccine dose in North Carolina;*** race was missing for 1.9%, and ethnicity was missing for 3.7% of vaccine recipients. Overall, 17.2% (95% CI = 17.1%–17.2%) and 5.6% (95% CI = 5.6%–5.6%) of vaccines were administered to Black and Hispanic persons, respectively. From December 14, 2020–January 3, 2021 to March 29–April 6, 2021, the proportion of vaccines administered to Black persons increased from 9.2% (95% CI = 9.1%–9.4%) to 18.7% (95% CI = 18.6%–18.9%) (p<0.001); during the same period, the proportion of vaccines administered to Hispanic persons increased from 3.9% (95% CI = 3.8%–4.0%) to 9.9% (95% CI = 9.8%–10.0%) (p<0.001) (Figure 2). During December 14, 2020–January 4, 2021, vaccination rates per 10,000 population aged ≥16 years were 66.2 and 72.6 for Black and Hispanic persons, respectively; during March 29–April 6, 2021, rates were 708.8 and 502.5 for Black and Hispanic persons, respectively. Among 57 counties that received vaccine allocations based on equity mapping, the proportion of Black persons vaccinated was equal to or higher than that of the Black population proportion in 11 (19.3%) counties, and equal to or higher than that of the Hispanic population proportion in five (8.8%) counties. By provider type, the proportion of persons who were Black or Hispanic vaccinated by FQHC providers exceeded their county population proportions by 6.5%, whereas non-FQHC providers under-vaccinated these groups by 2.5%.

**FIGURE 2:**
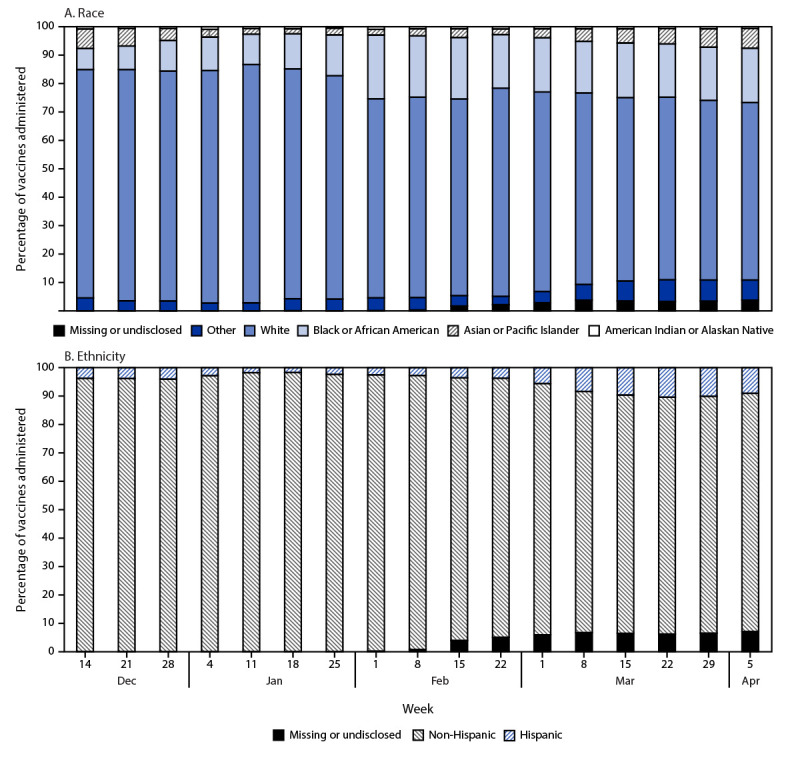
Percentage of COVID-19 vaccine doses (n = 2,815,774) administered, by race (A) and ethnicity (B)* and week^†^ — North Carolina, December 14, 2020–April 6, 2021 * Race and ethnicity self-reported by vaccinated persons and recorded by vaccine providers in the North Carolina COVID-19 Vaccination Management System. Data include all persons aged ≥16 years. ^†^ “Apr 5” week was 2 days (April 5–6, 2021); all other weeks were 7 days.

## Discussion

During December 14, 2020–April 6, 2021, implementation of a vaccine equity strategy coincided with a near doubling in the proportion of vaccine doses administered to Black persons aged ≥16 years in North Carolina, approaching parity with the population proportion of Black persons. The share of vaccine doses administered to Hispanic persons doubled during this period. The combination of strategies that might have helped promote vaccination among Black and Hispanic communities in North Carolina included mapping, promoting shared accountability with providers for equitable vaccine distribution through public dashboards and individualized performance reporting, and building partnerships to support vaccine access. Cumulative disparities in vaccine distribution among Black and Hispanic persons in North Carolina reflect similar disparities across the United States, which result in part from structural inequities that have affected health care access and trust in health care by these communities (*6*,*7*).

Mapping first focused on identifying the locations of residences of eligible Black and Hispanic persons aged ≥65 years and was then updated to focus on areas of lowest vaccination coverage among these groups. Mapping required complete data on race and ethnicity of vaccinated persons. After implementation of biweekly performance reporting for vaccine providers, North Carolina achieved a level of high completeness of race and ethnicity data, missing information for 5.1% of vaccination recipients (*3*) compared with 42.8% nationwide (*8*). Performance reporting also supported shared accountability by enabling providers to compare their performance, which might have motivated improvement. Finally, NCDHHS recognized the central role of partnerships with FQHC and community-based organizations in earning trust among Black and Hispanic communities. FQHC administered vaccines more equitably compared with other provider types. Community-based organizations, often in partnership with NCDHHS, hosted webinars to share vaccine information and listen to community needs.

NCDHHS continues to build on the strategy outlined in this report. On April 26, 2021, NCDHHS published a map of census tract-level vaccination coverage among persons aged ≥12 years^†††^ and Social Vulnerability Index^§§§^ (Supplementary Figure, https://stacks.cdc.gov/view/cdc/107719).^¶¶¶^ As of April 2021, state-contracted providers continue to establish vaccination clinics in census tracts with high social vulnerability indices and low vaccination coverage, and partners use the map to identify areas for conducting outreach. NCDHHS supports door-to-door health education and community mobilization through Healthier Together,**** a public-private partnership to increase vaccination coverage. Over 400 community health workers (>30% of whom are Spanish bilingual) provide culturally sensitive programming and support vaccination events.

The findings in this report are subject to at least four limitations. First, this ecological study did not include a comparison group, and observed changes cannot be attributed to the strategies implemented. Second, not all dimensions of vaccine equity were addressed. For example, vaccine recipient income and occupation were not recorded, which precluded assessments for these aspects of equity and socioeconomic status. Third, vaccination administration by race and ethnicity was not publicly tracked separately among persons aged ≥65 years, who were the focus of mapping strategies given their earlier eligibility and higher risk for severe COVID-19–related outcomes. Finally, this report does not describe all local equity-promoting strategies pursued by vaccine providers.

Equitable COVID-19 vaccination is critical to addressing the disproportionate incidence of COVID-19 among Black and Hispanic communities that are economically and socially marginalized (*1*,*9*). Multiple approaches are warranted to promote equitable distribution of vaccines (*10*). To prioritize equitable COVID-19 vaccinations among Black and Hispanic communities, NCDHHS used mapping, promoted shared accountability with providers for equity, and facilitated partnerships with community organizations to support vaccine access. These strategies could also be considered by public health officials in other states and communities to further increase equity in COVID-19 vaccine distribution and coverage, including among racial and ethnic populations disproportionately affected by COVID-19.

**Summary**

**What is already known about this topic?**

COVID-19 has disproportionately affected Black or African American and Hispanic communities.

**What is added by this report?**

Among persons vaccinated during March 29–April 6, 2021, compared with December 14, 2020–January 3, 2021, in North Carolina, the proportion who were Black nearly doubled, and the share of vaccine doses administered to Hispanic persons doubled during this period, approaching the proportion of the state population for these groups aged ≥16 years.

**What are the implications for public health practice?**

To promote equitable vaccination coverage, public health officials could consider using U.S. Census tract-level mapping to guide vaccine allocation, promote shared accountability for equitable distribution of vaccines with providers through data sharing, and facilitate community partnerships to support vaccine access.

## Footnotes

* Race and ethnicity were self-reported by vaccinated persons and recorded by health care providers in the NCDHHS COVID-19 Vaccination Management System (CVMS). Race and ethnicity were analyzed separately. Black persons were those who identified as being of Black race regardless of ethnicity. Hispanic persons were those who identified as being of Hispanic ethnicity regardless of race.

† Census tract-level population data were from the U.S. Census Bureau American Community Survey (2019 5-year estimates). North Carolina health care provider locations were from CVMS and the Federal Retail Pharmacy Program.

§ Based on data from CVMS.

¶ NCDHHS COVID-19 Response Dashboard: Vaccinations (https://covid19.ncdhhs.gov/dashboard/vaccinations).

** Current versions of toolkits are available in English and Spanish online at Supporting North Carolina’s Vaccination Efforts Toolkit for Partner Organizations (https://covid19.ncdhhs.gov/media/1482/download) and COVID-19 Vaccine Communications Toolkit (https://covid19.ncdhhs.gov/vaccines/covid-19-vaccine-communications-toolkit).

†† 45 C.F.R. part 46, 21 C.F.R. part 56; 42 U.S.C. Sect. 241(d); 5 U.S.C. Sect. 552a; 44 U.S.C. Sect. 3501 et seq.

§§ Data on AI/AN were not analyzed in this report because many vaccines were administered by the Indian Health Service, which was outside of NCDHHS purview. Asian or Pacific Islander (A/PI) persons were not the focus of the NCDHHS strategy because the proportion of persons vaccinated who were A/PI persons was at least equal to the population proportion in North Carolina from the start of vaccine rollout.

¶¶ Statewide population estimates were 2019 Bridged-Race Population estimates from the National Center for Health Statistics. https://wonder.cdc.gov/bridged-race-v2019.html

*** This report does not include vaccinations administered by federal vaccine providers, such as the Veterans Administration or the Indian Health Service.

††† Vaccination coverage was defined as the percentage of persons aged ≥12 years who were fully vaccinated (i.e., had received all recommended doses of a COVID-19 vaccine).

§§§ The CDC Social Vulnerability Index ranks census tracts according to 15 social factors (indicators). This metric helps to identify communities that might need additional support during emergencies, including the COVID-19 pandemic. https://www.atsdr.cdc.gov/placeandhealth/svi/documentation/SVI_documentation_2018.html

¶¶¶
https://nc.maps.arcgis.com/apps/webappviewer/index.html?id=31df85b470ad49809445a2d83e80d269

****
https://covid19.ncdhhs.gov/vaccines/nc-vaccine-strategy/healthier-together-health-equity-action-network
